# Supplementary material for: Evaluation of an On-Site Disaster Medical Management Course in Nepal
Source: Healthcare (Basel). 2024 Jun 30;12(13):1308. doi: 10.3390/healthcare12131308 (PMC11241171; doi:10.3390/healthcare12131308)
Supplement: Supplementary file 1 [file healthcare-12-01308-s001.zip › Supplementary Table S1.pdf]

Supplementary Table S1. Schedule of three-day Basic On-Site Disaster Medical Support Course.

Time	Topic	Type of Activity
**DAY 1**		
0830 hr	Course Registration	
0900 hr	Welcome and Introduction	
0905 hr	Disaster Site Medical Support Pre-test	Test Administration
0915 hr	Definitions and Abbreviations	Lecture
0920 hr	The Disaster Management Cycle	Lecture
0940 hr	Types and Effects of Disasters in Nepal	Lecture
1000 hr	Reinforcing Basic Facts about Disasters	Small Group Session
1010 hr	Planning for Disasters	Lecture
1030 hr	TEA BREAK	
1045 hr	Disaster Emergency Services and their Roles	Lecture
1115 hr	Briefing on Medical Support Planning Scenario	Lecture
1125 hr	Practical Training: Medical Support Planning with Disaster scenario	Small Group Session
1205 hr	Presentation of Medical Support Plans	Group Presentations
1245 hr	LUNCH	
1330 hr	Organisation of the Disaster Site	Lecture
1400 hr	Medical Support at Disaster Site	Lecture
1430 hr	Rapid Quiz on Medical Support at Disaster Site	Small Group Session
1445 hr	Organisation and Components of a First-Aid Post	Lecture
1500 hr	Ambulances, Stretchers, Stretcher Bearers	Lecture
1515 hr	TEA BREAK	
1530 hr	Practical Training: Setting up a First Aid Post	Small Group Session
1715 hr	Feedback and Instructions for Day 2	
1730 hr	Close of Day 1 Programme	

**DAY 2**		
0830 hr	Registration of Participants	
0900 hr	Activation of Medical Services during a Disaster	Lecture
0920 hr	Practical Training: Activation of Medical Services	Small Group Session
0950 hr	Presentation of Activation and Mobilization Plans	Class Presentation
1015 hr	Triage in Disasters	Lecture
1030 hr	TEA BREAK	
1045 hr	Triage Practical Exercise	Group Quiz Session
1130 hr	Disaster Communications	Lecture
1145 hr	Disaster Communications Exercise	Small Group Session
1230 hr	LUNCH BREAK	
1330 hr	Psychological Support in Disasters	Lecture
1400 hr	Forensic Support in Disasters	Lecture
1430 hr	Practical Training: Disaster Site Medical Command	Small Group Session
1500 hr	Disaster-site Medical Logistics	Lecture
1520 hr	Practical Training: Priority 1 Scenarios	Small Group Session
1550 hr	TEA BREAK	
1605 hr	Practical Training: Priority 2 Scenarios	Small Group Session
1630 hr	Practical Training: Priority 3 Scenarios	Small Group Session
1650 hr	Practical Training: Ambulance Point Scenarios	Small Group Session
1715 hr	Feedback and Instructions for Day 3	
1730 hr	Close of Day 2 Programme	

DAY 3		
0830 hr	Registration of Participants	
0900 hr	Coordination of Disaster Medical Services in Community	Lecture
0930 hr	Disaster Site Support in Floods	Lecture
0950 hr	Disaster Site Support in Fires	Lecture
1010 hr	Disaster Site Support in Earthquakes	Lecture
1030 hr	TEA BREAK	
1045 hr	Disaster Site Support in Landslides	Lecture
1105 hr	Disaster Site Support in Chemical Disasters	Lecture
1125 hr	Practical Training in Decontamination Techniques	Small Group Session
1235 hr	Debrief on Special Disaster Situations	Group Quiz Session
1245 hr	LUNCH BREAK	
1330 hr	Theory post-test on Disaster Site Medical Support	Test Administration
1400 hr	Moulage Training for Disaster Exercises	Lecture + Practical
1500 hr	Disaster Exercises Organisation, Conduct and Debrief	Lecture
1600 hr	Umpiring and Feedback Checklists for disaster-site exercises	Lecture
1630 hr	Practical Exercises in Disaster Scenario Planning	Small Group Session
1700 hr	Presentation of Disaster Scenario Plans	Class Presentation
1720 hr	Feedback and Assignments for Participants	
1730 hr	Close of Day 3 Programme	
