# Supplementary material for: Evaluation of an On-Site Disaster Medical Management Course in Nepal
Source: Healthcare (Basel). 2024 Jun 30;12(13):1308. doi: 10.3390/healthcare12131308 (PMC11241171; doi:10.3390/healthcare12131308)
Supplement: Supplementary file 1 [file healthcare-12-01308-s001.zip › Supplementary Table S2.pdf]

Supplementary Table S2: Organisational Affiliations of Participants

	Number of Participants (n=135)
**Healthcare Institutions/Hospitals**	
Nepalgunj Medical College Teaching Hospital	63
Koshi Zonal Hospital	30
Bheri Zonal Hospital	6
Neuro Cardio and Multi Specialty Hospital	5
Koshi Health Institute	4
Nobel Medical College Teaching Hospital	4
Birat Medical College	2
Biratnagar Aspatal	2
Nepal Sainik Hospital	2
Kaushalya Memorial Hospital	2
Amit Memorial Hospital	2
Birat Nursing Home	1
Nuwakot District Hospital	1
Nepal Police Hospital	1
**Non-Governmental Organisationss**	
Nepal Red Cross	7
Kohalpur Lions Club	1
**Ministry of Health and Population**	2
